# Computational and Experimental Evaluation of the Stability of a GLP-1-like Peptide in Ethanol–Water Mixtures

**DOI:** 10.3390/pharmaceutics14071462

**Published:** 2022-07-14

**Authors:** Lok Hin Lui, Raphael Egbu, Thomas Graver, Gareth R. Williams, Steve Brocchini, Ajoy Velayudhan

**Affiliations:** 1 UCL School of Pharmacy, University College London, London WC1N 1AX, UK; lok.lui.14@ucl.ac.uk (L.H.L.); raphael.egbu.16@ucl.ac.uk (R.E.); thomas.graver.17@ucl.ac.uk (T.G.); g.williams@ucl.ac.uk (G.R.W.); s.brocchini@ucl.ac.uk (S.B.); 2 Department of Biochemical Engineering, University College London, London WC1E 6BT, UK

**Keywords:** molecular dynamics, ethanol, stability, peptide, GLP-1 agonist, dimer formation, protein–protein interactions

## Abstract

Aggregation resulting from the self-association of peptide molecules remains a major challenge during preformulation. Whereas certain organic solvents are known to promote aggregation, ethanol (EtOH) is capable of disrupting interactions between peptide molecules. It is unclear whether it is beneficial or counterproductive to include EtOH in formulations of short peptides. Here, we employed molecular dynamics simulations using the DAFT protocol and MARTINI force field to predict the formation of self-associated dimers and to estimate the stability of a GLP-1-like peptide (G48) in 0–80% aqueous EtOH solutions. Both simulation and experimental data reveal that EtOH leads to a remarkable increase in the conformational stability of the peptide when stored over 15 days at 27 °C. In the absence of EtOH, dimerisation and subsequent loss in conformational stability (α-helix → random coil) were observed. EtOH improved conformational stability by reducing peptide–peptide interactions. The data suggest that a more nuanced approach may be applied in formulation decision making and, if the native state of the peptide is an α-helix organic solvent, such as EtOH, may enhance stability and improve prospects of long-term storage.

## 1. Introduction

With over 70 clinically used peptides and 150 in development, peptide therapeutics constitute a highly significant class of medicines [1]. Morbidities being successfully managed by polypeptide-based drugs include diabetes, for which glucagon-like-polypeptide (GLP-1) agonists are a key medication class [2]. From a drug development perspective, there are major challenges presented by peptide therapeutics, of which aggregation is one. Aggregation is a process where peptide molecules associate through both covalent and noncovalent interactions and form larger aggregates and become less soluble. These large-molecular-weight species can result in the loss of desired conformation of the therapeutic agent [3]. When exposed to conditions such as agitation and high temperature, peptides are capable of undergoing a specific type of β-sheet rich aggregation termed fibrillation [3]. Peptides in their aggregated state are nonefficacious and pose clinical toxicity risks [4,5]. The self-assembly of peptide particles is kinetically driven by nucleation events. Minimising or delaying nucleation events would improve the shelf life of peptide products. Thus, the viability of a peptide as a drug product is reduced by aggregation, leading to a significant interest in understanding aggregation and developing strategies to reduce aggregation during storage. Formulation approaches to reduce aggregation are primarily focused on proteins [6], and similar methods for peptides are sparsely discussed [7].

GLP-1 agonists exhibit an α-helical conformation [8]. In general, α-helices are unstable as monomers and exhibit a tendency to form more stable coiled-coil-like helices through association with other α-helices as dimers [9]. The associations of two or more peptide molecules is the first step towards nucleation. Hydrogen bonds between the backbone amide groups determine the α-helix and β-sheet secondary structure of peptides. The use of solvents that may disrupt hydrogen bonding may help to modulate peptide aggregation propensity. However, the exposure of peptides to organic solvents, such as DMSO [10] and acetonitrile [11], has been implicated in promoting aggregation. Exposure to such solvents may however be inescapable as solvents are often required for product development, for instance, in the formation of long-acting microspheres [10]. Unlike DMSO and acetonitrile, there are mixed reports about alcohols which have been observed to promote aggregation in some cases [12,13] but not others [14].

Monohydric alcohols, such as methanol (MeOH), ethanol (EtOH), and trifluoroethanol (TFE), have been studied extensively in their stabilising effect of α-helices [15,16,17,18]. Given their lower dielectric constant than water, monohydric alcohols are known to weaken intraprotein hydrophobic interactions within a tertiary structure while strengthening electrostatic interactions including intrapeptide backbone hydrogen bonds, improving the stability of secondary structures [15,16]. There are also theories suggesting self-clustering of alcohol molecules on protein surfaces, which could also play important roles in stabilising α-helical structures and preventing interpeptide interactions [17,18]. Chang and coworkers used NMR to compare the aggregation of native GLP-1 in pure water and in 35% TFE [19]. Their study demonstrated that GLP-1 undergoes extensive aggregation in pure water. When the same aggregated GLP-1 is diluted in 35% TFE, higher-order GLP-1 aggregates can be disrupted, and the resulting GLP-1 structure gives identical signals with those GLP-1 monomers, except for a small extension of the N-terminal α-helical structure observed [19]. These unique properties may be useful in maintaining the stability of α-helical peptides by reducing intermolecular associations between α-helices while hydrogen bonds within the α-helix remain stable. Both MeOH and EtOH are commonly used as solvents in the pharmaceutical industry. According to ICH Q3C, MeOH is listed as a class 2 solvent with a permitted daily exposure of 30 mg/day, and EtOH is classified as a class 3 solvent, which is a less toxic, with a permitted daily exposure of 166.7 mg/day [20]. Given that EtOH is often consumed in social settings, it is considered the safest solvent among monohydric alcohols.

As the particular solvent compatible with or required for the development of each peptide varies, an in silico method for determining aggregation propensity is an attractive option as it can support experimental studies to probe protein–protein interactions at the molecular level. All-atom molecular dynamics (MD) simulations offer a computational modelling approach to complement experimental work and gain important insights into molecular events occurring. All-atom MD simulations with explicit solvation are usually much preferred as they provide detailed information for protein aggregation. The simplest way to simulate protein aggregation using MD simulation is to place multiple copies of the protein of interest in the same simulation box and allow them to form protein complexes during the simulation. The first known all-atom MD simulation to study the aggregation of protein was reported by Klimov and Thirumalai in 2003 to study the assembly of the fragment of Alzheimer’s Aβ, Aβ_16–22_, over 40 ns of trajectories. [21] In order to speed up the aggregation kinetics in the MD simulation, they employed a harmonic coupling between the oligomer centre of mass and the centre of the water box [21]. As molecular dynamics codes and computing power continue to evolve over the years, later reported studies allow free assembly of peptides, but the total simulation time and replicates are limited [22,23,24]. Due to a significant computational cost associated, it remains prohibitive to simulate systems with two protein molecules for a meaningful simulation time using an all-atom force field to cover an entire energy landscape as a number of dimer species may be viable and disassociation events may not be observed in a single long simulation potentially due to low disassociation rates [25]. Therefore all-atom MD simulations are usually limited to studying the early or final stages of protein aggregation. To improve sampling in MD simulations, coarse-graining (CG) is often employed to reduce the degree of freedom in MD simulations by using simplified representations. As the CGMD approach significantly reduces the computational demands, replicates or long time-scale simulations of large and complex systems can be performed. MARTINI is a popular CGMD force field for simulating biomolecular molecules, including proteins. It has been shown to have high transferability, allowing quick adaption to different protein systems [25,26,27,28]. The MARTINI force field adapts a four-to-one mapping approach to reduce computational demand and is originally parameterised based on the reproduction of experimental partitioning free energies [27]. In particular, MARTINI provides suitable CG modelling methods for studying interactions with the α-helix [28,29]. Self-assembly of peptides has also been explored with the MARTINI model under various protein-formulation-relevant conditions, such as concentration, temperature, and pH [30,31,32,33]. Frederix and coworkers conducted a screening of all possible dipeptide combinations to predict aggregation propensities and nanostructure formations [33].

Using a single long simulation to study dimer formation is not deemed to be feasible as a large number of dimer structures may be present. Once a dimer complex has formed in the MD simulation, high free energy barriers may prohibit the disassociation of dimer complexes; hence, using a single long trajectory to study the formation of protein complexes from their monomeric state is deemed insufficient [29]. A large number of shorter MD simulation replicates where monomers are placed at noninteracting distances with random initial orientations can be used as an efficient alternative method to observe molecular arrangement and orientation in dimer formation. A docking assay for transmembrane components (DAFT) has been used to aid in setting up multiple MARTINI CGMD simulations with multiple molecules at noninteracting distances and identify peptide–peptide interactions and binding orientations [29]. This approach was originally used to study the formation of a glycophorin A helical transmembrane domain dimer in phospholipid bilayers [29]. Our group has previously used the same methodology to study antibody–antibody interactions in combination with virtual screening to guide the discovery of a specific excipient to inhibit aggregation [34].

The DAFT workflow uses a Python script, martinize, to convert an atomistic structure to MARTINI representations [35]. Subsequently, two peptide molecules are added with random relative orientations at predefined distances in a simulation box, solvated, and ionised with a Python script, insane [29]. The prepared systems are also compatible with martinate for streamlining energy minimisation, position-restrained isochoric−isothermal (NVT) equilibration, and production run [29].

This study employs a dual computational/experimental approach to study the dimerisation of an α-helical GLP-1-like peptide (G48). We have sought to understand the influence of EtOH on the conformational stability of G48 and to determine correlations between experimental and in silico data. The key aims are to:

(i) Provide insight into the potential stabilisation effect of solvents and, where the organic solvent is a known aggregator but inescapable;
(ii) Narrow the choice to the least aggregating candidate.

## 2. Materials and Methods

### 2.1. Docking Assay for Transmembrane Component (DAFT) Setup

There is no published structure of G48 available, so an α-helical model of the 29-residue structure of G48 was generated through homology modelling using CHARMM-GUI from a closely related structure [36], exendin-4 (PDB ID:1JRJ) [37]. The topology for G48 was generated with martinize [35]. The termini of G48 were considered neutral and patched accordingly. Five different conditions were considered: 0% EtOH, 20% EtOH, 40% EtOH, 60% EtOH, and 80% *v*/*v* EtOH in water. The DAFT script was used to prepare all CGMD dimerisation simulations of G48 in a MARTINI 2.2 protein force field. Each set of simulations contains 1024 replicates. Each system contains two G48 molecules placed at the same initial distance of 3.5 nm and randomly rotated, yielding different relative starting orientations. Each system is initially neutralised with 4 NA+ ion beads and solvated in a solvent box of roughly 8600–8660 standard MARTINI water beads in size, with box dimensions of approximately 12.80 × 12.80 × 6.20 nm^3^ and an average density of 999.0 kg/m^3^. In general, the MARTINI model is based on a four-to-one mapping of heavy atoms. The nonpolarised MARTINI water model was used throughout this work; in this, four water molecules are represented by a single uncharged P4 bead, and a single EtOH molecule is represented by a single P2 bead [25]. MARTINI water beads were substituted directly with MARTINI EtOH beads on a one-to-one basis using a bash script to modify the solvent environment to the corresponding ratio of EtOH.

### 2.2. Molecular Dynamics Simulations

After the simulation systems were prepared, the martinate script was used to manage system energy minimisation and system equilibrations with GROMACS 2016.3 (University of Groningen, Groningen, The Netherlands) [38], which consists of minimisation at 1 fs/step for 500 ps and NPT equilibration at 20 fs/step for 100 ps. Finally, the CGMD simulations were performed at 20 fs/step for 512 ns with snapshots taken every 25000 steps. Periodic boundary conditions were used to simulate an infinite system. Temperature was kept constant with a V-rescale thermostat [39] at 300 K, and a Berendsen barostat [40] was used to maintain pressure at 1 bar. The 300 K temperature was chosen based on the original temperature used in the MARTINI parameterisation [27], and this temperature was also used in later experimental studies. Nonbonded interactions related to electrostatic and van der Waals interactions were computed using a potential-shift-verlet as modifier at a cut-off distance of 1.8 nm.

The use of coarse-grained molecular dynamics in studying peptide–peptide interactions may be questionable as it has been reported that the MARTINI force field can cause excessive aggregation [41]. Unlike those biologically important protein–protein interactions that mediate biological functions, pharmaceutically optimised proteins may not have any distinctive self-interacting hotspots. While the current protocol may not be able to accurately measure the magnitude of dimerisation, the 1024 replicates allow both specific and random intermediate orientations to be simulated, so the dimerisation landscape can be established. Therefore, this supports the validity of using the MARTINI force field with the DAFT protocol for studying self-interactions of pharmaceutical peptides.

### 2.3. Computational Analyses

#### 2.3.1. Interaction Energies

The nonbonded interpeptide interaction energy profiles were predicted by calculating the sums of Lennard–Jones and Coulomb potentials between all pairs of beads belonging to different peptide molecules within the MARTINI CG model using the GROMACS energy tool. In these coarse-grained simulations, the interaction energies become negative as the two simulated peptides interact with each other, and they were plotted as time vs energy. The calculated plateau values from the mean interaction energy distributions in different solvent systems were calculated with nonlinear least squares (NLS) fitting of the data to an NLS decay model, performed using R as described in the original DAFT protocol [29].

#### 2.3.2. Orientation Analysis

Relative orientations of the two G48 molecules in each set of conditions were characterised by calculating the Euler angles using the doriana tool [29]. The Euler angles were calculated from the rotation matrix corresponding to the least squares fit of a reference structure. In order to evaluate only the interacting dimers in the simulations, only snapshots with the sums of Lennard–Jones and Coulomb potentials of two G48 peptides below 0 kJ/mol were evaluated. Euler angles and the sums of Lennard–Jones and Coulomb potentials were plotted as a two-dimensional kernel density estimation with R.

#### 2.3.3. Interaction Pattern

To identify the residues mediating peptide dimerisation under different conditions, the final snapshots were projected with VMD 1.9.3 (University of Illinois Urbana-Champaign, Urbana, IL, USA) [42] by aligning the backbone of one molecule and coloured in grey, with the relative locations of the BB (backbone) beads of the second molecule projected as red dots. The distance of all backbone beads between the two simulated G48 molecules in all trajectories within a set of simulations was calculated using the GROMACS tool mindist to describe the closest interacting point within a G48 dimer structure. The backbone bead pair with the shortest distance in each frame was identified. The occurrence of the closest backbone bead pair in each frame was further processed with the Matlab hist3 function to generate a bivariate histogram plot for the visualisation of the most common interacting bead pairs under each condition.

#### 2.3.4. Fluctuation in the Structure

To analyse the local structure fluctuation after exposure to the solvent environment, the final snapshots of each set of 1024 individual simulations were pulled together, and a total of 2048 poses of G48 were obtained. The structure of G48 was analysed using root mean square fluctuation (RMSF) to quantify the local structural fluctuation and intrapeptide motions based on the average position of the MARTINI BB beads. The helical parameter was calculated using the HELANAL Python script within the MDAnalysis package [43,44]. Local bending angles were calculated as the angle between the axes of the helical cylinders formed by four consecutive MARTINI backbone BB beads, following the method of Sugeta and Miyazawa [43]. The window of four MARTINI BB beads slides along the length of the G48 peptide. The angles between successive local axes can be used to determine local bends (<20°) or kirks (>20°).

### 2.4. Experimental Stability Studies

G48, provided by AstraZeneca (Cambridge, UK), is a polypeptide containing 29 amino acids (AA). The molecular weight and isoelectric point (PI, based on ionisable groups) of G48 are 3.5 kDa and ~5, respectively. Stock solutions of the peptide were prepared in water at a concentration of 5 mg/mL (adjusted to pH 7.4). Pure analytical-grade EtOH (>99.9%) was added to the peptide solution and gently mixed such that the final solution (500 µg/mL) contained one of 0%, 20%, 40%, 60%, or 80% EtOH. The mixture was pipetted into lyophilisation vials, which were stoppered (Wheaton, Millville, NJ, USA) and then further sealed with parafilm to prevent evaporation. The vials were incubated at 300 K and, at predefined intervals, retrieved for characterisation.

#### 2.4.1. Circular Dichroism

A Chirascan Plus spectrometer (Applied Photophysics, Leatherhead, UK) was used to obtain the circular dichroism (CD) spectra. UV (400–230 nm) and CD (260–200 nm) analyses were conducted with 0.5 and 10 mm Quartz Suprasil rectangular cells (Starna Scientific Ltd., Ilford, UK), respectively. The measurements were conducted at 300 K with the following parameters applied: 1 s accumulation time per point, 1 nm spectral bandwidth, and 1 nm stepwise. Diluent (ethanol and water) and light scattering correction were applied to all measurements. Normalisation to account for concentration and cell path length was also applied to the far-UV CD spectra. The results are presented in mean residue ellipticity (MRE, deg·cm^2^·dmol^−1^) according to Equation (1):

(1) $$\mathrm{MRE}=\frac{{\mathrm{MRW}*\theta }_{\mathrm{obs}}}{10*l*C}$$

$\theta_{\mathrm{obs}}$ denotes the ellipticity in millidegrees, C and l represent concentration and pathlength, and MRW is the mean residue weight. An APL Prodata Viewer (Leatherhead, UK) was used for data processing. The secondary structure distribution was estimated using the BeStSel secondary structure prediction analysis tool [45].

#### 2.4.2. Quantification of Soluble Peptide

Using an 1100 HPLC instrument (Agilent, Cheadle, UK) equipped with a Supelco Biowide C18 column (4.6 mm × 150 mm × 5 µm), the quantification of the amount of soluble peptide was undertaken at predefined intervals. At the defined intervals, supernatants of the centrifuged (5 min, 4000 RPM) samples were diluted with water prior to analysis. The eluents were HPLC-grade (A) water with 0.1% trifluoroacetic acid (TFA) and (B) acetonitrile with 0.1% TFA. The following gradient method was applied: 0–4 min 70% (A) 4–5.5 min linear gradient to 40% (A) 7.5–9.5 min linear gradient of (A) up to 70%, followed by a washout period of 3.5 min. Each measurement had a 13 min total run time. UV absorbance at a wavelength of 214 nm and a retention time of 7.9 min was used to determine the peptide content.

## 3. Results

### 3.1. In Silico G48 Dimerisation

#### 3.1.1. Interaction Energies

**Figure 1:**
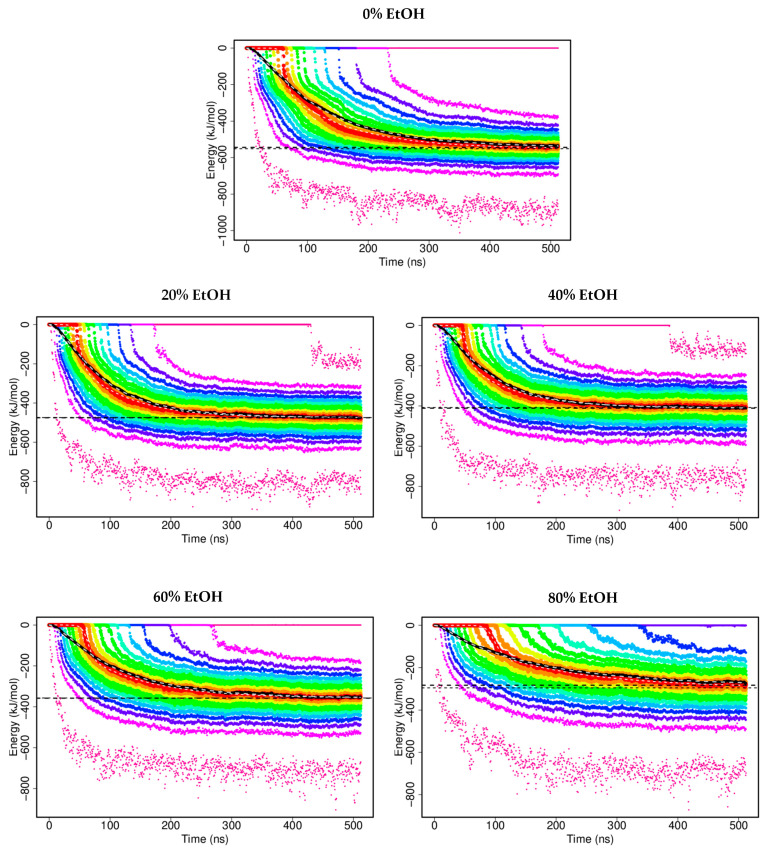
The distribution of interaction energies (sum of Lennard–Jones and Coulomb interactions) between G48 dimers over time in 0–80% *v*/*v* EtOH. Each colour represents 5% quantile increments of interaction energies. The black-dotted line shows the plateau values calculated using an exponential decay model. The interaction energy between two G48 monomers solvated in higher concentrations of EtOH is less negative compared with those in lower concentrations of EtOH. At 80% EtOH, dimerisations of G48 were not observed in around 15% of simulations.

The formation of G48 dimers was explored with the aid of DAFT using MARTINI CGMD simulations in five different solvent environments, 0%, 20%, 40%, 60%, and 80% *v*/*v* EtOH in water. Each condition yielded 1024 individual trajectories, and the time evolution of the interaction energy (sum of Lennard–Jones and Coulomb interactions) between two G48 monomers was analysed and plotted as distribution vigintiles with 5% quantiles (Figure 1). At the beginning of simulations, two molecules of G48 were separated, and the interaction energies were minimal. As the simulation progresses, the G48 molecules diffuse freely in the MD simulation system and may encounter one other. The interaction between two G48 molecules shifts the interaction energies to negative. However, not all aqueous EtOH simulations show dimerisation of G48. In the case of 0% EtOH, about 5% of simulations did not show any interactions before 512 ns, while around 15% of simulations for 80% EtOH did not dimerise during the simulation time. The final mean interaction energy of G48 dimers was reported as the calculated plateau values from mean interaction energy distributions (Table 1).

#### 3.1.2. Interacting Points

**Figure 2:**
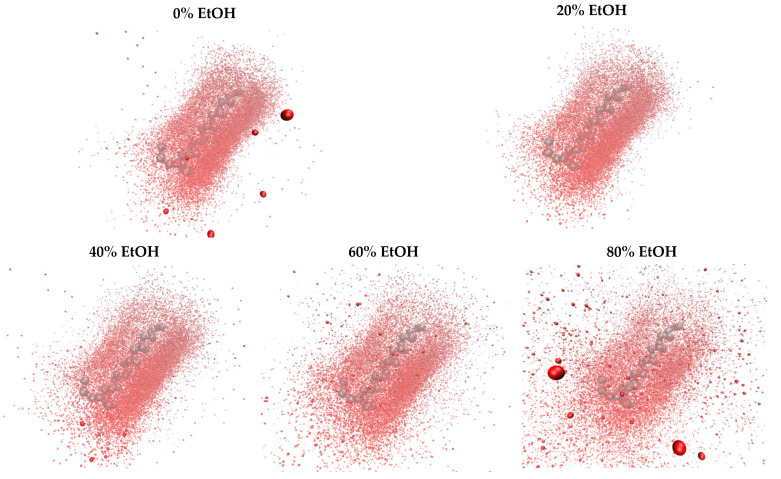
The dimer structures are visualised by aligning the coordinates of the first molecule in the final snapshots to show the distributions of the backbone (BB) beads of the second molecule around the first molecule. The first molecule is coloured grey with the N-terminal located on the left, and the distributions of the relative positions of the BB beads of the second molecule are coloured red. In the absence of EtOH, the G48 dimers are formed mostly along the helical axis. As the concentration of EtOH increased, a more diverse dimer formation pattern was observed.

The coordinates of the first G48 molecule final snapshots have been aligned to aid visual comparisons of the dimer structures. The distributions of the backbone (BB) bead of the second G48 molecule around the first molecule are shown in Figure 2, providing an overview of the final dimer conformations in different EtOH solutions. In the case of 0% EtOH, the BB beads from the second molecule are densely populated around the first molecule. The distribution of BB beads around the N-terminal is sparse, suggesting that the G48 N-terminal does not contribute to dimerisation.

**Figure 3:**
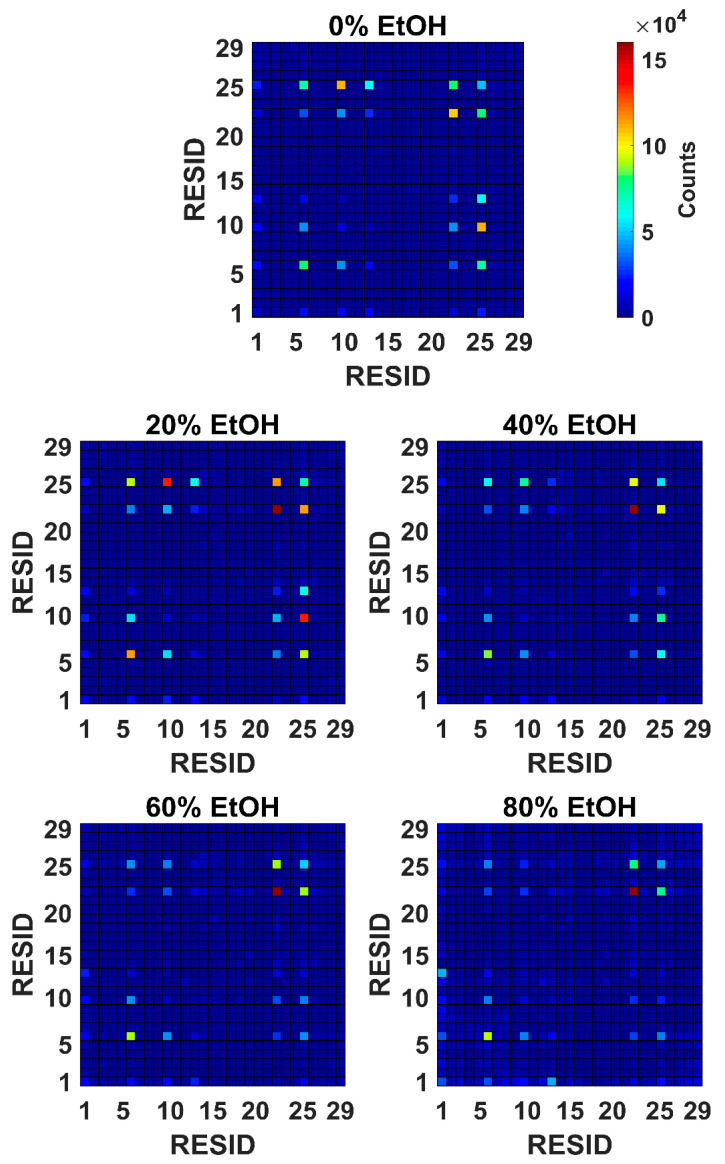
The most common interacting backbone bead pairs in a set of 1024 MARTINI CG simulations with two molecules of G48. Five distinct interaction points were observed, PHE6, TYR10, TYR13, PHE22, and TRP25. The number of distinctive interaction pairs reduces as the concentration increases, with PHE6-PHE6, PHE22-PHE22, and PHE22-TRP25 remaining at 80% EtOH.

GROMACS mindist calculates the distance between any two pairs of beads from the two different G48 molecules for all frames within a simulation set, and the most common interaction pairs were identified (Figure 3). Five distinct interaction points were observed in the case of 0% EtOH. These are PHE6, TYR10, TYR13, PHE22, and TRP25. With the addition of EtOH at 20%, an increase in interactions was seen between all of these five interaction points. A further increase in the proportion of EtOH to 40% led to a reduction of interactions between TYR10 and TYR13 with TRP25. At 60% and 80% EtOH, the only distinctive interaction pairs remaining were PHE6-PHE6, PHE22-PHE22, and PHE22-TRP25 with dimerisation interactions through PHE22 becoming more common.

#### 3.1.3. Orientation Analysis

**Figure 4:**
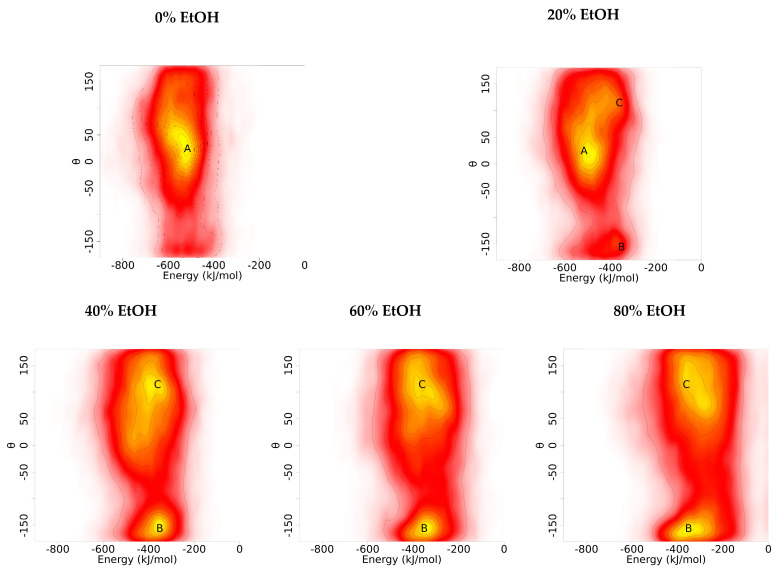
Relative orientations are described as the tilt angle (θ). Population densities are represented by contoured colours, where white to red indicates low densities and yellow represents high densities. Parallel dimers A are predominately found in 0% and 20% EtOH. Antiparallel dimers B and C were found in all conditions containing EtOH.

To characterise the orientation of G48 dimers in different EtOH concentrations, Euler angles were calculated and analysed for each simulation set. For each condition, the orientation was described with the tilt angle (θ) (Figure 4). A tilt angle between −50° and 50° indicates parallel dimerisation, and a tilt angle above 130° or below −180° suggests antiparallel dimerisation. The main cluster (A) observed in 0% EtOH has tilt angles of approximately 20° with estimated interaction energies of around −530 kJ/mol. Cluster A was also observed in 20% EtOH but not at higher EtOH concentrations. At 40% EtOH or above, cluster B was the primary cluster found under these conditions. Cluster B was also found in 20% EtOH to lower the extent. Cluster B consists of antiparallel dimers with tilt angles close to −155°, and their estimated interaction energies were −350 kJ/mol, which was lower than those of cluster A. Besides clusters A and B, an additional cluster (C) was identified as the second-largest cluster in all conditions containing EtOH. Cluster C shows tilt angles in the region of 110° with interaction energies of approximately −390 kJ/mol.

#### 3.1.4. G48 Stability

**Figure 5:**
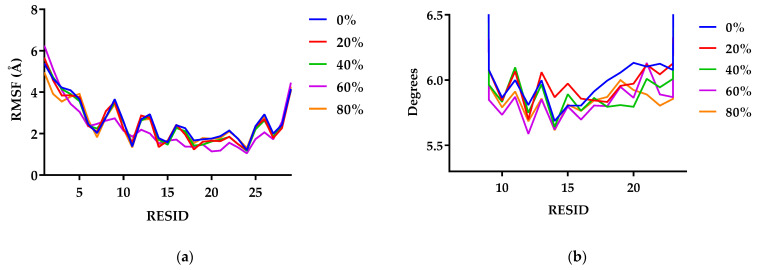
RMSF values (**a**) and local bend angle (**b**) per residue in G48 in EtOH compositions of 0–80% *v*/*v*. The α-helical structure of G48 was stable in different EtOH concentrations with no kirks identified in the core G48 structure.

The conformational stability of G48 in different EtOH compositions was analysed by both RMSF and HELANAL based on the final configurations of G48 in each of the 1024 trajectories. The shapes of the RMSF profiles of a backbone residue in the five tested conditions are similar (Figure 5a). In general, the overall mobility of residues within the α-helix core (residues 7–27) in 0% EtOH is slightly larger than that in the EtOH–water mixtures; hence, the intrapeptide motions are greater without EtOH. For the majority of residues, systems with EtOH fluctuate less than the system without EtOH.

Local bend angles were calculated with HELANAL within MDAnalysis, and helical axes were calculated by using the coordinates of four contiguous backbone atoms (Figure 5b). Analysing the local bend angles shows that the α-helical structure of the peptide has local bends of less than 6.5° with no kirks identified in the core G48 structure across all five conditions. In the second half of the α-helical structure (residues 17 to 22), the local bend angles for G48 solvated in water are higher than those with EtOH mixture.

### 3.2. Experimental Results

#### 3.2.1. Secondary Structure of G48

**Figure 6:**
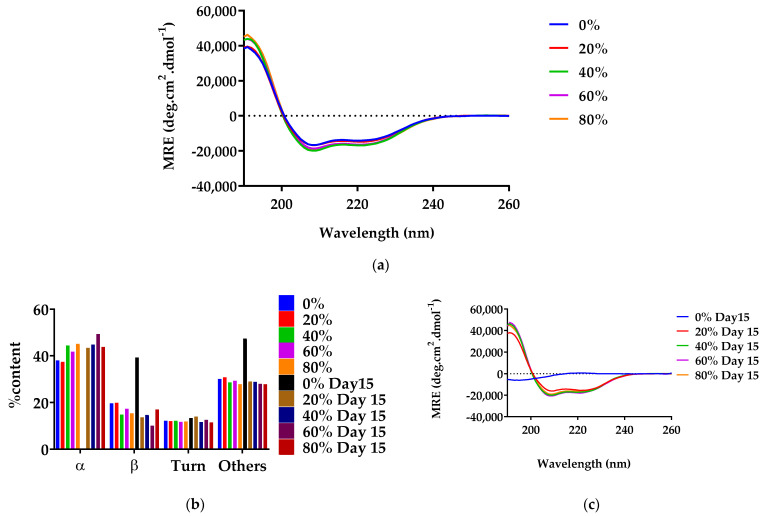
Secondary structure of G48 stored at 0–80% *v*/*v* EtOH, determined by far UV CD. (**a**) Day 0, (**b**) day 15, (**c**) a breakdown of the conformations as determined by the BeStSel secondary structure analysis tool [45]. Significant G48 structural deformation in 0% EtOH was seen from day 15 onwards, whereas G48 structural integrities were maintained in all other conditions with EtOH.

Other GLP-1 agonists, such as exendin-4, are known to possess a primarily helical structure in their native state [8]. As depicted in Figure 6a, G48 possessed similar helical conformation to exendin-4 on day 0 with minima at 208 and 224 nm in the presence or absence of EtOH.

Figure 6b indicates that the inclusion of EtOH in G48 solution led to maintenance in the α-helical conformation at 27 °C even after storage for 15 days. As such, the minima at 208 and 224 nm remained evident. This was observed for all of the aqueous EtOH solutions. There was slightly more reduction in the minimum at 208 nm in 20% EtOH than at other values. Conversely, on day 15 with no EtOH present, there was a shift in minima to 195 nm, which is attributable to the conversion of the initial α-helical conformation to random coils. Additionally, noticeable was a reduction in ellipticity magnitude, which is attributable to the loss in soluble peptide. The proportion of α-helical content in G48 solutions was elucidated from the CD data using BeStSel secondary structure analysis [45].

On day 0, G48 existed primarily in an α-helical conformation in solution (Figure 6c). The α-helical content remained relatively constant in the presence of 20–80% EtOH. After storage for 15 days, the α-helix content in the 0% EtOH preparation was completely absent, and instead, there was a marked rise in ‘other’ conformations, which included random coils attributable to the minima at 195 nm (Figure 6b) mentioned earlier and loops and bends. There was also an increase in the β-sheet content. Conversely, in the presence of EtOH at all percentage values, negligible change in the α-helix content was observed for the 15-day duration investigated. The data show that whereas G48 lost its stability when stored at 0% EtOH, the inclusion of EtOH solution was capable of aiding the peptide in maintaining its conformational stability above room temperature for 2 weeks.

#### 3.2.2. Soluble Peptide Quantification

**Figure 7:**
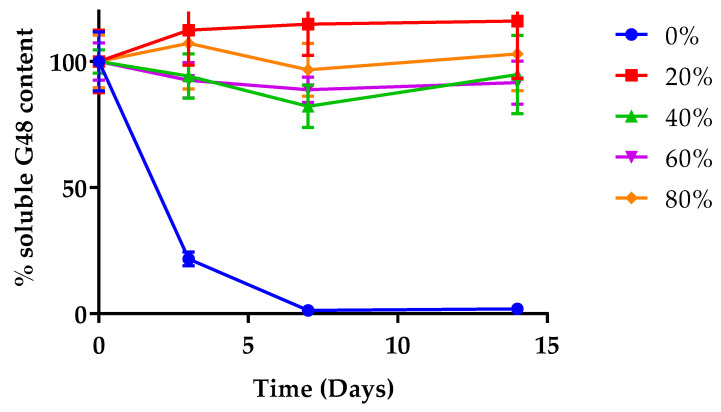
Quantification of soluble G48, determined by HPLC. The samples were stored for up to 15 days following the addition of 0–80% *v*/*v* EtOH solution. An almost complete loss of soluble G48 stored in 0% EtOH was seen from day 7 onwards, whereas the solubility of G48 was maintained at higher concentrations of EtOH.

HPLC was utilised for the quantification of soluble G48 present in the solutions containing 0–80% EtOH. Figure 7 depicts a complete loss of soluble G48 content by day 7 with 0% EtOH.

The data corroborate information obtained from CD (Figure 6), where the reduction in ellipticity magnitude is likely attributable to the reduction of soluble G48. In stark contrast, EtOH aided the preservation of G48 in solution state across the 15 days investigated in this study such that G48 content remained at around 100% on day 15.

## 4. Discussion

Peptide storage remains a major challenge owing to their propensity to aggregation, often requiring refrigeration (2–8 °C) to maintain stability in the clinic [46]. To the authors’ knowledge, there are no reports in the literature on the incorporation of EtOH into formulation of GLP-1 agonists. Furthermore, the literature is lacking with regard to the influence of EtOH on the storage of peptides. The majority of reported cases describe immediate or medium-term effects of organic solvents on proteins [13,15]. Previous studies carried out on newt fibroblast growth factor (nFGF_1_) looked at the influence of 40–75% EtOH on aggregation [11]. Here, we expanded on that range (0–80% EtOH) in the study of peptide G48 and complemented our findings with simulation data in order to derive a mechanistic insight into the processes occurring.

When two simulated peptides interact with each other, the sum of Lennard–Jones and Coulomb potentials becomes negative. A decrease (more negative) in calculated interaction energies of the attractive interactions between two G48 peptide molecules refers to an overall increase in interactions between two peptide molecules, which can be useful to predict aggregation propensities. The in silico data show a large variation of the interaction energies ranging between 0 and −1000 kJ/mol, indicating that a large number of dimer configurations are possible for G48. Dimerisation in 0% EtOH displayed the most negative interaction energies among all tested conditions. Wassenaar and coworkers [29] carried out studies on the formation of glycophorin A helical transmembrane domain dimers in phospholipid bilayers (GpA-WT and GpA-G83I) with the DAFT workflow. The interaction energies for the simulations for G48 in 0% EtOH were more negative than the cases of both GpA-WT and GpA-G83I. However, when G48 was solvated at higher ratios of EtOH/water, the calculated plateau value became less negative between G48 molecules. The addition of EtOH also reduced the spread of interaction energies. These plateau values suggest that the G48 dimers formed in 0% EtOH are more thermodynamically stable, and the energy barrier for the dissociation of G48 dimers in 0% EtOH is higher and more prone to aggregation. Soluble dimer formation is likely the first step towards nucleation and subsequent aggregation. Reducing dimerisation in the presence of EtOH should therefore increase the lag time for aggregation. Around 15% of simulations for 80% EtOH did not dimerise in this in silico study compared with simulations for 0% EtOH. Eighty percent EtOH reduced the formation of dimers and prevented interactions between monomers. The occurrences of self-interactions at the same residues increased at higher concentrations of EtOH, so it is reasonable to suggest that EtOH can reduce intermolecular interactions between G48 monomers.

As the concentration of EtOH increases, the distributions of backbone beads of the second peptide molecule around the first peptide molecule become more scattered, showing a more varied pattern of self-interactions with less intense interpeptide interactions. The total interacting surface areas were decreased, and the interaction energies were also reduced as there were fewer beads involved in the dimerisation. This provides an alternative view on why the number of simulations that resulted in different dimer configurations in 0% EtOH, 20% EtOH, 40% EtOH, and 60% EtOH and the calculated plateau value for the interaction energies are less negative as the concentration of EtOH increases. As only around 85% of simulations for 80% EtOH resulted in a dimer configuration, the distributions of the BB beads are more scattered, reflecting those noninteracting simulations.

The orientations of G48 dimers in 0% EtOH were predominately parallel (Cluster A), and these G48 dimers within cluster A had interaction energies similar to the plateau values calculated from the mean interaction energy distributions. Cluster A was also found at 20% EtOH but not at higher EtOH concentrations. At 40% EtOH or above, cluster B was predominant, and these dimer configurations are thought to be antiparallel, which has slightly more positive interaction energies. The secondary cluster, cluster C, found in all conditions with EtOH has a tilt angle of around 110°, which is considered neither parallel nor antiparallel; these dimer complexes are almost at a right angle to each other, giving larger total surface areas compared with parallel and antiparallel dimers. Thus, this further explains why the distributions of the BB beads are more scattered at higher concentrations of EtOH as these configurations are not as compact as parallel or antiparallel dimers. These results also suggest that the addition of EtOH would prevent the formation of more thermodynamically stable parallel dimers, and the antiparallel dimers in cluster B are more favourable at higher concentrations of EtOH. Since the interaction energies of these antiparallel dimer configurations are less thermodynamically stable, it is reasonable to suggest that these antiparallel dimers are less likely to aggregate.

All of the five amino acid residues responsible for interpeptide interactions are hydrophobic residues (PHE6, TYR10, TYR13, PHE22, and TRP25). The polarity of EtOH is lower than that of water, and thus, it is possible that the hydrophobic side-chain residues interact preferentially with EtOH rather than the hydrophobic side chain of the residues of neighbouring peptide molecules [15]. Both RMSF and local bend angle calculations suggest that the addition of EtOH does not destabilise the structure of G48. Self-interaction between G48 molecules at PHE22 is more common at higher concentrations of EtOH, whereas a larger range of residues act as interaction points for systems solvated in 0% EtOH. Hence, the local fluctuations in the α-helical structure are reduced as the number of possible dimer conformations is reduced.

To complement the data obtained from simulation, CD was utilised. As peptides are optically active, they possess the capability of absorbing circularly polarised light. Interpreting this absorbance of light at certain wavelengths can elucidate the conformation of peptides [47]. The CD data clearly show that the addition of an aqueous EtOH solution maintained the helical conformation of the peptide for 2 weeks when stored outside the fridge (27 °C). Typically, the conversion of monomers to aggregates is accompanied by a reduction of soluble peptides [46]. Chromatographic data tracking the loss in soluble peptide complemented the CD data in showing physical instability over time in the absence of EtOH. The experimental data, however, did not fully match the positive linear relationship between % EtOH and G48 content, which was obtained in silico. The reason for this is likely the inability to detect such subtle differences with experimental techniques, such as HPLC, and/or the small changes in concentration that may have resulted from the evaporation of EtOH. Notwithstanding, to prevent evaporation, the samples were stored in lyophilisation vials, appropriately stoppered, and then further sealed with parafilm. Nonetheless, the simulation data indicate a reduction in the formation of thermodynamically stable parallel dimers and overall dimerisation with increased ethanol concentrations and, hence, a reduction in aggregation, and these hypotheses are supported by experimental data showing reduced aggregation on a gross scale.

While organic solvents, such as methylene chloride [13], DMSO [10], and acetonitrile [11], have been shown to accelerate aggregation, alcohols have a tendency to enhance helicity and so may be relevant stabilisers in cases where the native state of the peptide is α-helical. Studies looking at the influence of alcohol on aggregation have mainly been carried out on larger peptides and proteins [11,48]. It is believed that the unfolding of quaternary and tertiary proteins to a helical structure results in nucleation-dependent aggregation and thus phenomena such as fibrillation [3]. This has been shown in insulin and nFGF_1_ [3,11]. Clearly, such a position does not apply to peptides such as GLP-1 agonists whose native conformation is α-helical, and where alcohols can promote formulation stability by reducing dimerisation, thus delaying nucleation. As such, solvents that do not directly affect degradation while maintaining helicity could provide formulation stability and be considered stabilisers from a formulation perspective. The data in our study have shown that EtOH maintained the native helical sate of the peptide G48. Similar observations were seen in an earlier study by our group using exendin-4, a 39-residue long GLP-1 agonist with a predominantly α-helical structure. The addition of EtOH reduces the degradation of exendin-4 compared with the formulation with water alone. An in silico study of exendin-4 dimerisation also indicated a decrease in interactions between molecules of exendin-4 in the presence of EtOH. Although the trend seen in our in silico model did not show in the experimental studies presented here, the reduction of interaction energies has indicated the right direction for aggregation reduction and offers a qualitative predictive tool for in silico formulation development.

## 5. Conclusions

We employed CGMD simulations using the DAFT protocol with the MARTINI force field for predicting the formation of self-associated dimers and estimating the stability of a GLP-1-like peptide (G48) in different compositions of EtOH. These simulations were completed with experimental work using circular dichroism and HPLC. The addition of EtOH to an aqueous G48 solution markedly improved the storage stability of the peptide, as evidenced by the maintenance of its α-helix conformation. This is achieved by reducing intermolecular hydrophobic interactions, resulting in different dimer configurations, while the stability of the α-helix backbone is unaffected. These intriguing results provide a rational approach for considering formulating peptides in EtOH.

## Figures and Tables

**Table 1:** The plateau values calculated from the mean interaction energy distributions. As the concentration of EtOH increases, the interaction energy (sum of Lennard–Jones and Coulomb interactions) between two G48 monomers is less negative. This suggests that the G48 dimers formed at a higher concentration of EtOH are becoming less thermodynamically stable compared with those formed at lower EtOH concentrations.

Solvent Environment	Plateau Value (kJ/mol)	Standard Error of the Mean
0% EtOH	−544.0	0.3
20% EtOH	−474.9	0.2
40% EtOH	−410.9	0.2
60% EtOH	−357.5	0.2
80% EtOH	−283.6	0.3

## Data Availability

All related data and methods are presented in this paper. Additional inquiries should be addressed to the corresponding author.
